# Evolution and differential expression of a vertebrate vitellogenin gene cluster

**DOI:** 10.1186/1471-2148-9-2

**Published:** 2009-01-05

**Authors:** Roderick Nigel Finn, Jelena Kolarevic, Heidi Kongshaug, Frank Nilsen

**Affiliations:** 1 Department of Biology, University of Bergen, Bergen High Technology Center, Postbox 7803, N-5020, Bergen, Norway; 2 Nofima Marine, N-6600 Sunndalsøra, Norway; 3 Institute of Marine Research, Post box 1870 Nordnes, N-5817 Bergen, Norway

## Abstract

**Background:**

The multiplicity or loss of the vitellogenin (*vtg*) gene family in vertebrates has been argued to have broad implications for the mode of reproduction (placental or non-placental), cleavage pattern (meroblastic or holoblastic) and character of the egg (pelagic or benthic). Earlier proposals for the existence of three forms of vertebrate *vtgs *present conflicting models for their origin and subsequent duplication.

**Results:**

By integrating phylogenetics of novel *vtg *transcripts from old and modern teleosts with syntenic analyses of all available genomic variants of non-metatherian vertebrates we identify the gene orthologies between the Sarcopterygii (tetrapod branch) and Actinopterygii (fish branch). We argue that the vertebrate *vtg *gene cluster originated in proto-chromosome m, but that *vtg *genes have subsequently duplicated and rearranged following whole genome duplications. Sequencing of a novel fourth *vtg *transcript in labrid species, and the presence of duplicated paralogs in certain model organisms supports the notion that lineage-specific gene duplications frequently occur in teleosts. The data show that the *vtg *gene cluster is more conserved between acanthomorph teleosts and tetrapods, than in ostariophysan teleosts such as the zebrafish. The differential expression of the labrid *vtg *genes are further consistent with the notion that neofunctionalized Aa-type *vtgs *are important determinants of the pelagic or benthic character of the eggs in acanthomorph teleosts.

**Conclusion:**

The vertebrate *vtg *gene cluster existed prior to the separation of Sarcopterygii from Actinopterygii >450 million years ago, a period associated with the second round of whole genome duplication. The presence of higher copy numbers in a more highly expressed subcluster is particularly prevalent in teleosts. The differential expression and latent neofunctionalization of *vtg *genes in acanthomorph teleosts is an adaptive feature associated with oocyte hydration and spawning in the marine environment.

## Background

A defining feature of the early development of non-eutherian vertebrates is a cleidoic egg endowed with variable amounts of yolk. Until very recently, the major products of the vitellogenin (*vtg*) genes that encode yolk proteins have been considered to be simple precursors of the energy reserve of vertebrate eggs, but the latest studies have demonstrated several non-nutritional roles for Vtg [1,2]. Similarly, the recent observation that remnants of *vtg *genes exist in Eutheria, including humans, but have sequentially been lost through co-evolution with casein genes [3] elegantly demonstrated that the three known *vtg *genes in birds represent a conserved gene complement. In a previous study, Finn & Kristoffersen [4] proposed a model for the evolution and neofunctionalization of *vtg *genes in acanthomorph teleosts. We identified *vtgC *as an ancestral gene, and argued that the dual *vtgAa/vtgAb *system, first noted by La Fleur et al. [5] was derived from a single form, the A-type *vtg*. In this model, the separation of the *vtgC*- and *vtgA*-type genes occurred following the second round (R2) of whole genome duplication (WGD). Subsequently *vtgA *duplicated and formed paragolous *vtgAa *and *vtgAb *genes in acanthomorph teleosts. This phylogenetic model has been corroborated by other investigators [6]. Most recently however, Babin [7] has provided a syntenic map of vertebrate *vtg *genes, which shows that the three forms of *vtg *are encoded in a *vtg *gene cluster (VGC) in non-eutherian vertebrates. A major goal of the present study was to integrate the statistical, biochemical and physical models of *vtg *gene evolution in vertebrates.

Through a series of studies, we and other laboratories have shown that the pelagic nature of a marine teleost egg is an evolved feature [4] that primarily results from the maturational influx of water due to differential degradation of VtgAa-type yolk proteins (Yp), and the temporal insertion of novel aquaporins (Aqp1b) in the microvillous portion of the plasmalemma [8-10]. The neofunctionalization of the *vtgAa *form in acanthomorph teleosts, has sensitized the heavy chain domain (LvH-Aa) to catheptic proteolysis that generates a large organic osmolyte pool of free amino acids (FAA) in the ovulated egg [1,9,11-19]. In contrast the LvH domains derived from *vtgAb *and *vtgC *genes may be partially cleaved, but remain mostly intact following the maturational proteolytic event, and thus contribute minimally to oocyte hydration [1,12,14,17,18]. In teleosts that spawn benthic eggs (benthophils), a character that we have argued to be the ancestral condition due to an ancient freshwater heritage (Finn & Kristoffersen, 2007), Yps may be cleaved or partially processed to generate a small pool of FAA during oocyte hydration [6,20-24].

In order to reconcile the differences in the phylogenetic, biochemical and syntenic models we have examined the evolution and expression of the *vtg *gene complement in modern (Perciformes: Labridae) and old (Clupeocephala: Clupeidae) teleosts that spawn pelagic and benthic eggs. We were interested in determining how the expression of different *vtg *transcripts relates to the character of the egg in a single family of closely related teleosts, and whether lineage-specific gene duplication resulted in neofunctionalization of the Aa-type *vtgs*. To determine the orthologies and ancestry of the novel labrid *vtg *genes cloned in the present study, we anchored the results of phylogenetic inference with the syntenic arrangement of genomic *vtgs *in model vertebrates. This approach allowed us to identify the proto-chromosomal origin of the VGC that is conserved between the Actinopterygii (fish branch) and the Sarcopterygii (tetrapod branch). It further allowed us to conclude that neofunctionalization of the *vtgAa *genes in acanthomorph teleosts occurred long after the duplication event.

## Results and discussion

### Multiple transcripts

**Figure 1:**
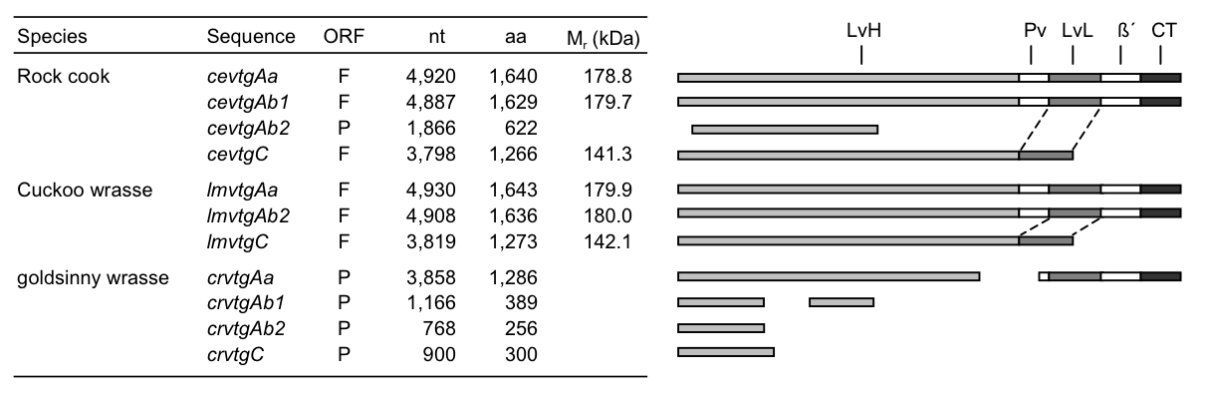
**Overview of the cloned labrid genes showing full (F) or partial (P) open reading frames (ORF) of the nucleotide (nt) and deduced amino acid (aa) sequences**. Linear representations of the sub-domain structures of each sequence are shown to the right.

A total of eleven *vtg *transcripts (six full-length and five partials) were cloned from vitellogenic livers of the labrid species investigated (Fig. 1). NCBI BLAST searches of the deduced proteins verified that all sequences are members of the Vtg family. Four distinct *vtg *cDNA sequences were cloned from rock cook (*Centrolabrus exoletus*) and goldsinny wrasse (*Ctenolabrus rupestris*), two of which (*cevtgAb1 *and *crvtgAb1*) were entirely novel for vertebrates. Three transcripts (*lmvtgAa*, *lmvtgAb2 *and *lmvtgC*) were obtained from cuckoo wrasse (*Labrus mixtus*). Repeated attempts using gene-specific primers to extract a second *vtgAb*-type transcript from livers of cuckoo wrasse did not yield any novel transcripts. The deduced amino acid sequences of the full-length rock cook and cuckoo wrasse Vtgs revealed that the VtgAa and VtgAb products are complete pentapartite type proteins (NH_2_-LvH-Pv-LvL-β'-CT-COO^-^) while VtgC belongs to the phosvitinless class of Vtg (NH_2_-LvH-LvL-COO^-^) [25].

**Figure 2:**
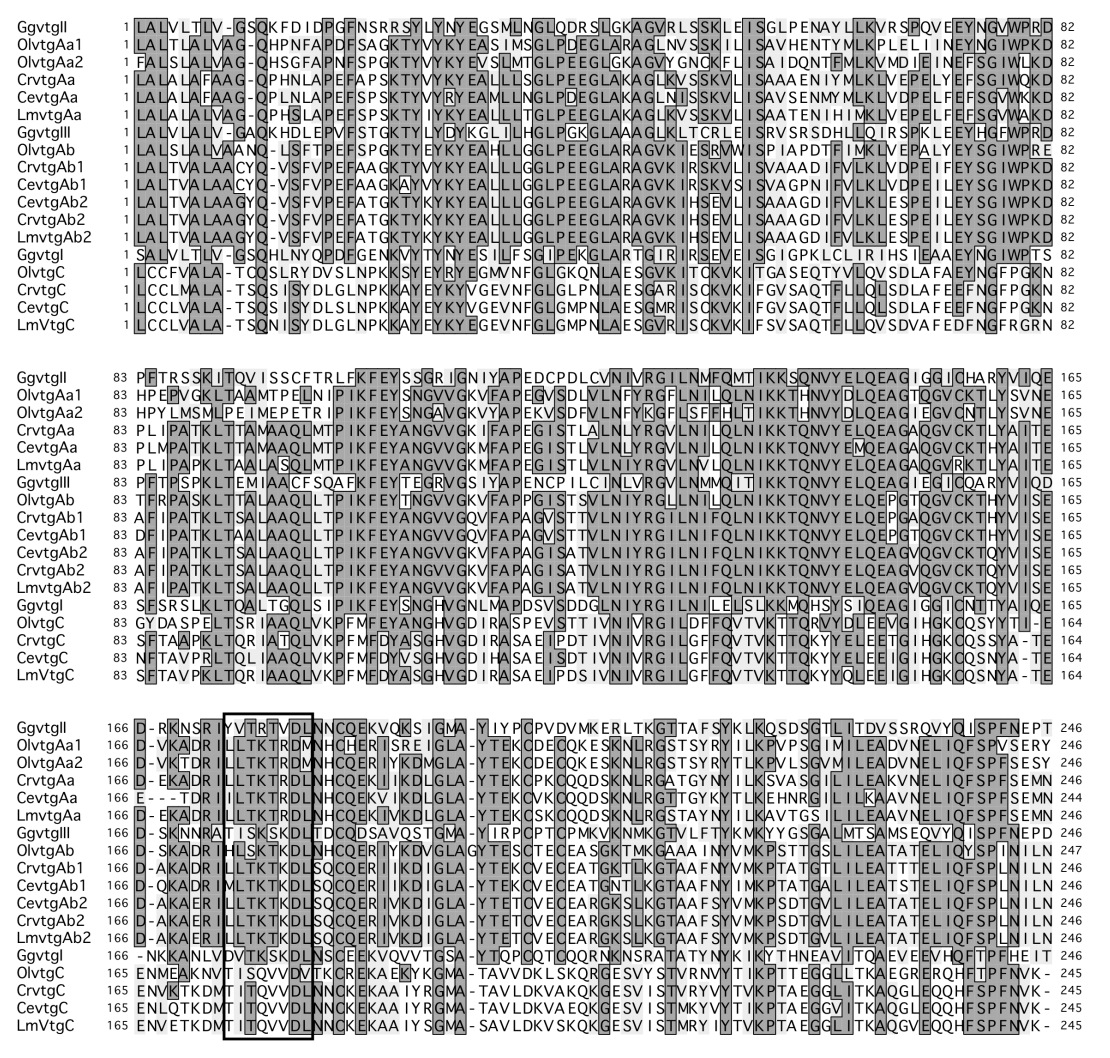
**Multiple sequence alignment of the conserved N-terminal region of labrid vitellogenins (Crvtg: goldsinny wrasse, Cevtg: rock cook; Lmvtg: cuckoo wrasse) in relation to expressed variants in chicken (GgvtgI, GgvtgII, GgvtgIII) and medaka (OlvtgAa1, OlvtgAa2, OlvtgAb, OlvtgC)**. Sequences are arranged according to orthology (VtgII/VtgAa, VtgIII/VtgAb, VtgI/VtgC). The boxed residues represent the Vtg receptor minimal interaction domain identified for tilapia by Li et al. [56]. See materials and methods for explanation of the novel OlvtgAa2 sequence, and Additional file 1 for accession numbers used in the study.

**Figure 3:**
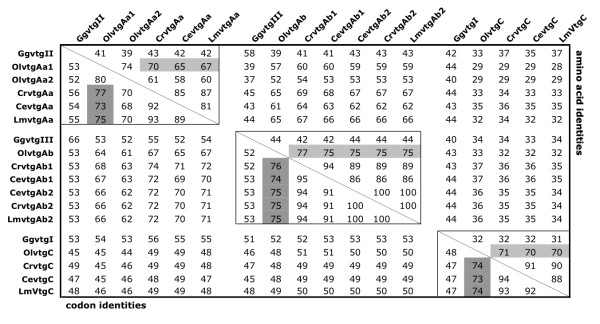
**Amino acid and codon identities of aligned labrid, chicken and medaka vitellogenins shown in Fig. **1.

Since it is now postulated that the vertebrate *vtg *gene complement represents a conserved cluster in Sarcopterygii (the tetrapod branch) and Actinopterygii (the fish branch) [7], we aligned the longest partial segment of the labrid sequences to the genomic variants in chicken (*Gallus gallus*) and medaka (*Oryzias latipes*), respectively (Fig. 2). Relative homology scores of the aligned amino acids and codons revealed three forms with highest identity to medaka *olvtgAa1*, *olvtgAb *and *olvtgC*, respectively (Fig. 3). The relationship between the teleost and chicken genes was less obvious, with essentially equal identity scores for the *ggvtgII *and *ggvtgIII *products. Interestingly, GgvtgI had slightly lower scores to the teleost C-type products compared to the Aa- or Ab-type products over the aligned LvH sub-region (Fig. 2). This suggests that despite their orthology (see below) the minor *ggvtgI *and *vtgC *genes have functionally diverged in association with the loss of the phosvitin (Pv) and C-terminal domains in teleosts.

Within the labrid species, sequences had highest identities to their homologs in closely related goldsinny wrasse. Since only a single *Ab*-type transcript was detected in cuckoo wrasse, and it showed 100% identity to the novel *cevtgAb2 *and *crvtgAb2 *sequences over the ~250 aligned aa and ~750 aligned nt (Fig. 3), we named the cuckoo wrasse *Ab*-type sequence *lmvtgAb2*.

**Figure 4:**
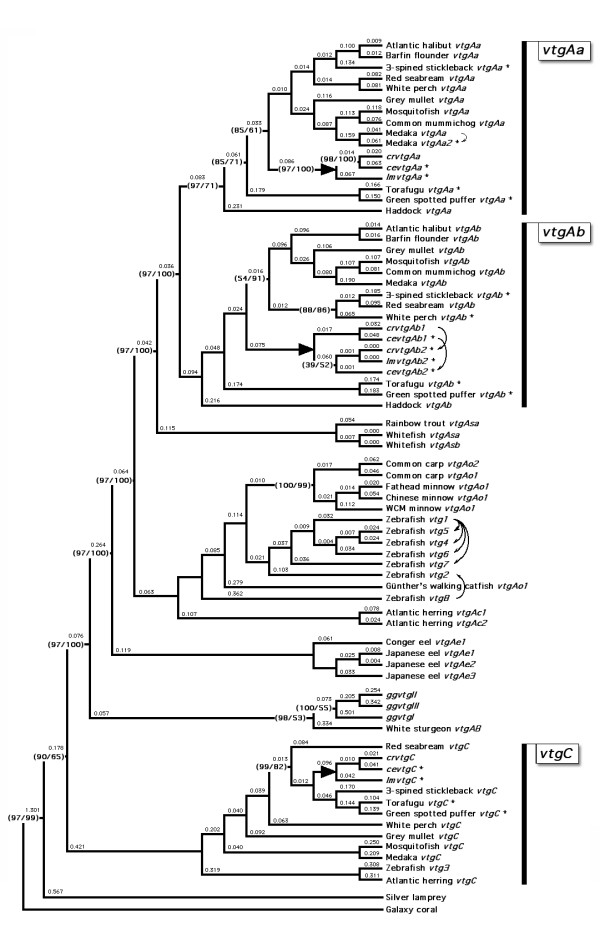
**Maximum likelihood tree of aligned vertebrate vitellogenin codons**. Novel sequences, including genomic variants are annotated with a "*". Arrows represent the duplication of genes outlined in Fig. 5. Numbers in brackets represent Bayesian posterior probabilities of codon and amino acid alignments, respectively. Unlabelled nodes had 100% posterior probabilities and 100% bootsrap values for Bayesian and parsimony analyses, respectively. Branch lengths represent estimates of the number of nucleotide substitutions per site.

To further classify the labrid *vtgs*, we conducted large-scale phylogenetic analyses of the deduced amino acid and codon alignments of available (genbank) and novel genomic (ensembl) variants. For the genomic variants, we identified three genes in 3-spined stickleback (*Gasterosteus aculeatus*), torafugu (*Takifugu rupribes*) and spotted green pufferfish (*Tetraodon nigriviridis*), respectively, and four genes in medaka, of which two have been independently sequenced (see Additional file 1). In zebrafish eight genes are found in the genome, of which several have been fully [26] or partially sequenced [27], and each of which is expressed and deposited in the growing oocyte [28]. Validation of the tree topology was achieved through multiple methods of phylogenetic inference. Each method consistently clustered the labrid sequences as three forms: *vtgAa*, *vtgAb *and *vtgC*, respectively (Fig. 4). For Bayesian, maximum parsimony and neighbour-joining analyses the majority of branches were supported by 100% posterior probabilities, and 100% bootstrap values, respectively, with only minor branch rearrangements within gene groups. These data verify that three forms of *vtg *exist with acanthomorph teleosts, two within protacanthomorph teleosts [29], and three within ostariophysan teleosts. The data further revealed that a fourth novel gene in medaka is an *Aa*-type product, and we thus classified it as *olvtgAa2*. In a separate study, we have identified three novel gene variants in a basal clupeocephalan teleost, the Atlantic herring (Kristoffersen et al. unpublished data). These Atlantic herring transcripts clustered as a basal node to all ostariophysan *vtgs *in full agreement with the clupeocephalan phylogenetic rank.

The present analyses thus fully corroborate our earlier study of vertebrate *vtgs *[4] wherein the major and minor transcripts cluster according to taxonomic group. We further verified the inferred duplication of the *vtgAa *and *vtgAb *clusters using the method of Zmasek and Eddy [30]. Hence the tree topology is inconsistent with the syntenic arrangement of vertebrate *vtg *genes, and suggests that different functions have evolved within the major clades. Specifically for acanthomorph teleosts, the LvH-Aa domains have evolved a sensitivity to acidic degradation following activation of V-class proton pumps during oocyte maturation [1,9,11-13,15,17,18]. The data for labrid teleosts that spawn benthic and pelagic eggs support this view [[19,20], see below].

In order to understand the disparity between the phylogenetic and syntenic arrangement of vertebrate *vtg *genes, we increased the phylogenetic data set to include all known sarcopterygian *vtgs *(108 sequences, containing ~370 k nt), including amphibian, bird, reptile and platypus variants. In addition we examined the syntenic positions of *vtg *genes within non-metatherian vertebrates. The larger data set did not affect the topology of the teleost branches, but did place *ggvtgI *and the partial platypus transcript (ENSOANT00000031211) at the base of the tetrapoda and closer to, but on a separate branch to teleost *vtgC *variants (data not shown). Similarly, two further partial platypus transcripts (ENSOANT00000008462 and a construct of ENSOANT00000013101-ENSOANT00000013100) clustered as a basal node to *ggvtgIII *and *ggvtgII*, but after amphibian variants. These findings agree with the recent studies of Brawand et al. [3] and Babin [7] who demonstrated that three genes exist in monotremata and are putative orthologs of the three bird genes. Since these latter platypus genes are not yet localized to any chromosome, but are annotated in contigs 49.51 and 49.49, respectively, it was not possible to discern their true orthology, or syntenic arrangement in relation to chicken. However, by combining the results of the present phylogenetic data with the syntenic positions of the chicken and teleost *vtgs*, it can be stated that teleost *vtgC *genes are the putative orthologs of *ggvtgI*, and that teleost *vtgAa *and *vtgAb *genes are the putative orthologs of *ggvtgII *and *ggvtgIII*, respectively.

Previously we showed that the evolution of Vtg sub-domains is neither clock-like, nor under strong functional constraint [4]. We further highlighted the disparate retention of the sub-domain structures of the mature proteins. Teleost VtgC proteins have all lost the highly phosphorylated polyserine Pv region and the C-terminal domains that are homologous to human VWFD. In chicken, however, all three Vtgs, including GgvtgI, are complete type proteins containing all encoded domains. The same appears to be true for other tetrapod Vtgs, exemplified by amphibia and the platypus, although Brawand et al. [3] have shown that premature stop codons or indels have led to loss of function in *ggvtgII/III *orthologs in the platypus. The loss of sub-domains in teleosts is not restricted to the VtgC class. Amongst all ostariophysan teleosts, which represent the second largest superorder of fishes [31-33], the major gene expressed encodes a truncated form of Vtg that lacks the Vwfd region [26,27,34-36]. However, a complete-type gene is present in zebrafish as *vtg2*, and a novel juxtaposed gene (*vtg8*) encodes a protein that lacks the Pv and CT domains, i.e. a tripartite protein (NH_2_-LvH-LvL-β'-COOH). To address the *vtg *orthologies in Ostariophysi, we thus included all known zebrafish coding variants in the phylogenetic data set and examined their loci.

**Figure 5:**
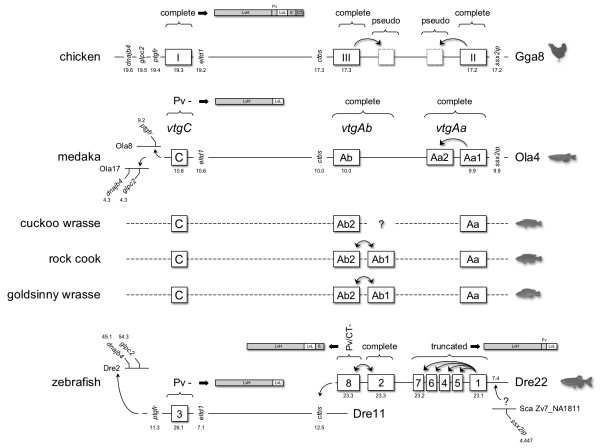
**Syntenic arrangement of chicken, medaka, labrid and zebrafish vitellogenin (*vtg*) genes**. White boxes represent coding *vtgs*, while dot-boxes indicate pseudogenes. Data for chicken show the chromosomal loci (Mb) of complete type *vtgs *together with some of the linked genes. Syntenic data for three-spined stickleback, torafugu and spotted green pufferfish are similar to the medaka [7], and therefore not shown here. The genes are aligned (not to scale) to illustrate orthologies between the vertebrates. Arrows infer the likely direction of duplication or rearrangement. Labrid *vtgs *are shown on dashed lines since no genomic data are currently available for linkage maps. The ZfVTG1-8 cluster is preliminary oriented according to increasing bp loci and a BLAST hit for the Ssx2ip protein. The rearrangement of the genes to different chromosomes is consistent with their origin in protochromosome m [see [40]]. Linear representations of the sub-domain structures of each type of Vtg are shown for clarity.

Unlike other acanthoperygian and sarcopterygian animals for which genomic data are available, the chromosomal loci of zebrafish *vtgs *are complex. Seven genes (*vtg1*, *vtg2*, *vtg4*, *vtg5*, *vtg6*, *vtg7 *and *vtg8*, hereafter called ZfVTG1-8) are located in a tight cluster on Dre22 (bp 23,098,595 – 23,301,096), while *vtg3 *is located on Dre11 (bp 26,086,024 – 26,105,006). However, none of the upstream or downstream genes that flank the *ggvtgII/III *or *vtgAa/Ab *clusters in sarcopterygians or teleosts, respectively, are currently annotated on Dre22. The ZfVTG1-8 cluster thus represents an island of *vtg *genes that has either lost the flanking genes, or translocated from another chromosome such as Dre11. Alternatively, the chromosomal loci may reflect the remnants of WGD, where differential loss via diploidization [37-39], or germline fusion and fission events have only left traces of the ancestral condition [40-42]. Indeed Kasahara et al. [40] have argued that Tni1 in spotted green pufferfish, which has retained the VGC, is derived from fusion of proto-chromosomes f, g and m, while Ola4 in medaka, which also has retained the VGC, is derived from proto-chromosome m. Interestingly medaka Ola17 which has lost the VGC, but retained the flanking genes, and Ola20 which also is derived from proto-chromosome m, as are Tni6 and Tni15 in spotted green pufferfish are the likely genomic remnants of the lost VGC paralogons. In zebrafish the derivatives of proto-chromosome m have translocated to Dre6, 8, 11 and 22, and fission variants have been retained in Dre2 and 24 [40]. Thus orthologs of the genes that flank the VGC in chicken should be localized in these chromosomes. This happens to be the case (Fig. 5). However, since none of the orthologs that flank the chicken VGC are annotated on Dre22, deciphering the orientation of the ZfVTG1-8 remains challenging. The synovial sarcoma, X breakpoint 2 interacting protein (Ssx2ip) that is juxtaposed to GgvtgII and OlvtgAa1, as in all other vertebrates [7], is currently annotated in scaffold Zv7_NA1811 in zebrafish. A BLAST search for this protein revealed multiple hits throughout the genome. However, one hit (e = 5.2 × 10^-6^) was found on Dre22 at position 7.4 Mb. We have therefore oriented the ZfVGC1-8 as shown in Fig. 5, which also corresponds to the increasing bp positions observed in other vertebrates.

To ascertain the local cis-duplication events, we integrated the results of the phylogentic analyses. The topology of the ZfVTG1-8 shown in Fig. 4 precisely replicates the chromosomal loci of each gene in the genome shown in Fig. 5. This is also true for the meadaka VGC, where *olvtgAa2 *represents an internal duplication of the independently sequenced *olvtgAa1 *(Q8UW88_ORYLA). In fact each of the major genes that are expressed in vertebrates are located at the outer edge of the *ggvtgII/III *and *vtgAa/Ab *VGC. This arrangement appears to have implications for the differential expression of the cluster, where cis-regulatory elements associated with estrogen induction [43,44] and recruitment of the transcription initiation complexes are concerned.

**Figure 6:**
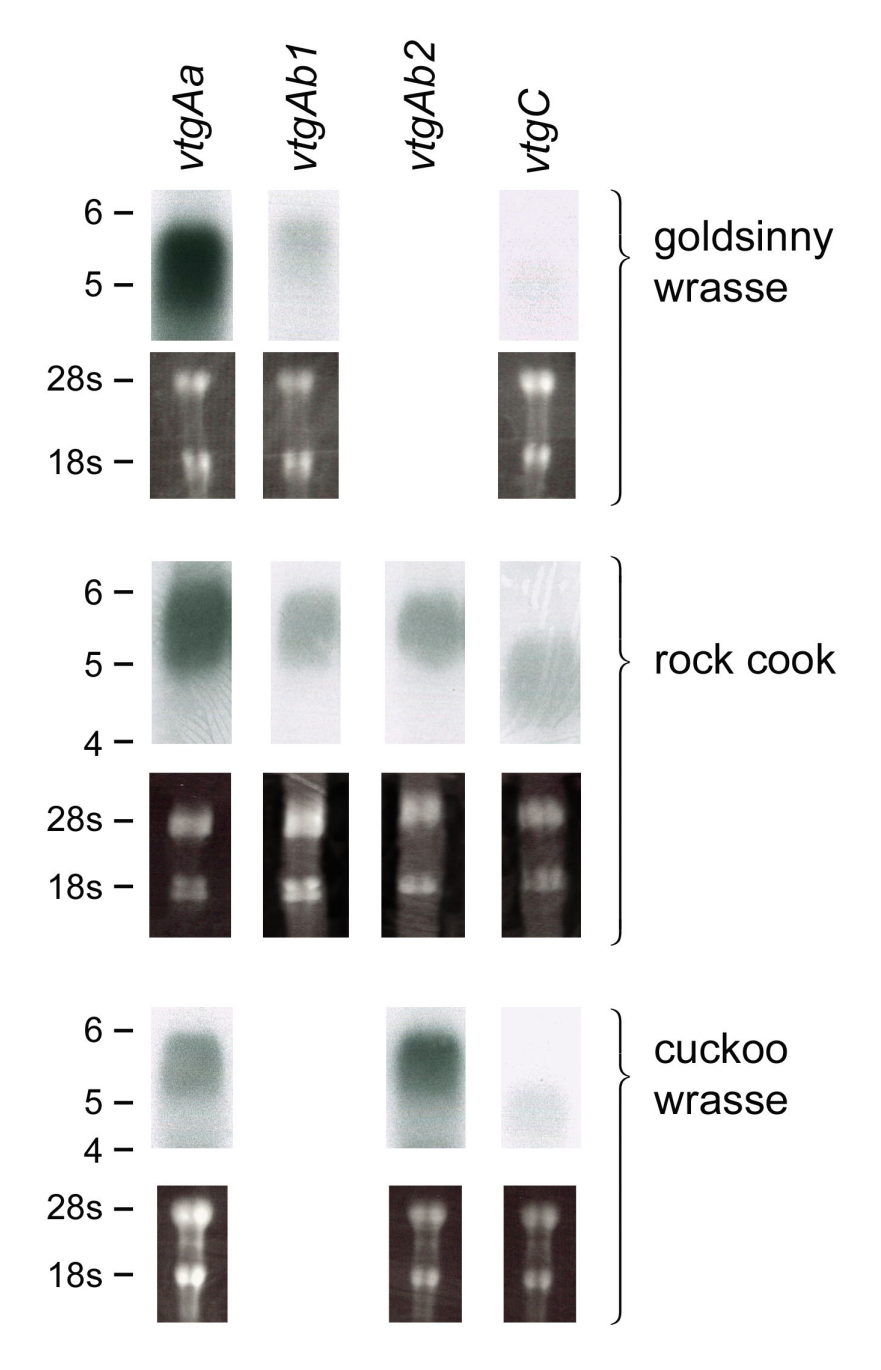
**Northern blots of the novel labrid vitellogenin transcripts**. Data were obtained from four *vtg *gene specific probes for rock cook and three for cuckoo and goldsinny wrasse ranging is size from 602 to 736 nt from the N-terminal regions. Total RNA loaded in each lane was ~3 μg μL^-1 ^and is visualized by 28 s and 18 s rRNA bands following ethidium bromide incubation and UV exposure. Markers to the left represent kbp.

In zebrafish, *vtg1 *is the major gene, which can be expressed at levels 100 – 1000× higher than other variants [27]. Similarly, in other teleosts, expression ratios of the *Aa*- or *Ab*-type *vtgs *vary according to species [1,12,24,25,45-47]. This is also true for the major *ggvtgII *gene in chicken [48]. Previously we have shown that in goldsinny wrasse, extreme levels of Aa-type Yps are incorporated in the growing oocyte [19]. These Yps are substantially degraded to FAA during pre-ovulatory maturation, leaving only a lipovitellin light chain (LvL-Aa) fragment for embryonic development. Due to the neofunctionalization of VtgAa [4], the degree of Yp proteolysis may depend upon the relative expression levels of each *vtg*, and it has been predicted that a dynamic expression ratio of *vtgAa *and *vtgAb *forms should exist in relation to ambient temperature and salinity, egg size and the presence of oil globules [25]. To investigate this possibility, we performed Northern blots of hepatically expressed transcripts in the three labrid species (Fig. 6).

The selected model of labrid teleosts are best known for their cleaning behaviour, a character that is currently being exploited as an environmentally-friendly means of biological control of ectopic parasites in mariculture. Members of this family, the third largest of vertebrates [49] with more than 600 species in 82 genera [50] are also known for their remarkable sex lives [51], where sex change, transvestitism and male-dominated parental care is prevalent. Although only one species spawns pelagic eggs in the temperate coastal waters of our Norwegian study area, it is noteworthy that all labrid teleosts are marine species, with the vast majority spawning pelagic eggs in a subtropical environment [52]. An understanding of the differential expression in the non-disrupted *vtg*-gene complement in this family of fishes is an important step towards development of sex-specific molecular markers for this group.

Probes were designed from the highly conserved N-terminal area of each cDNA sequence and their gene specificity verified by subsequent cloning and sequencing. With the exception of *crvtgAb2*, a single band of relevant size was detected after hybridization in each species. Although several levels of regulation lie between an expressed hepatic transcript and deposited Yp product in the growing oocyte, it is noteworthy that *vtg *band intensities were highly correlated to the type of Yp deposited in the oocytes and the pelagic or benthic nature of the spawned egg. For the goldsinny wrasse, which spawns pelagic eggs and generates the largest pool of FAA during oocyte hydration, an exceptional level of *vtgAa *is expressed in the liver compared to the other *vtg *transcripts. These data strongly support our earlier findings where virtually all of the oocyte Yps are maturationally proteolysed to FAA that subsequently drive the osmotic flow of water into the highly hydrated egg [19]. In the benthophil rock cook, more even band intensities of the four *vtg *transcripts are found, although higher levels of *vtgAa *are also expressed in this species. The rock cook is a close relative of the corkwing wrasse (*Crenilabrus melops*), which generates a small pool of FAA from partial Yp proteolysis during oocyte hydration [20]. We have recently conducted a proteomic analysis of the oocyte and egg Yps in the rock cook and cuckoo wrasse and found that moderate oocyte hydration is associated with proteolysis of mainly *vtgAa *derived Yp products (Kolarevic unpublished data). In cuckoo wrasse, a species that spawns benthic eggs and shows very limited maturational proteolysis, higher levels of VtgAb-type Yps are deposited in the oocyte. These latter observations are further supported by the fact that although greater amounts of RNA were inadvertently loaded in the *vtgAa *lane, the *vtgAb2 *band had the highest intensity. The significance of higher expression levels of Ab-type *vtgs *relates to the fact that these genes have not neofunctionalized [4] and their Yp products remain mostly intact in the hydrated egg as the major protein reserve for the developing embryo. Taken together, these data are in line with the notion that differential expression of non-neofunctionalized and neofunctionalized *vtgs *in acanthomorph teleosts is related to the benthic or pelagic character of the spawned egg.

## Conclusion

We find that labrid teleosts differentially express up to four *vtg *genes that are orthologous to an ancient *vtg *gene cluster that existed prior to the separation of Actinopterygii from Sarcopterygii. With the exception of zebrafish, the vertebrate *vtg *gene cluster remains linked on single chromosomes that arose in close association with the second round of whole genome duplication (WGD) >450 million years ago. Our model for lineage-specific duplication of the major *vtg *genes in teleosts shows that they comprise a variable subcluster. The copy number of this variable subcluster, which comprises the *ggvtgIII*/*vtgAb *and *ggvtgII*/*vtgAa *orthologs, is likely to be the combined result of the third round of WGD in teleosts with subsequent gene loss due to chromosomal rearrangements followed by lineage-specific gene duplications. The topology of the phylogenetic tree for the 8 zebrafish *vtg *genes precisely replicates their chromosomal loci in the genome and suggests that lineage-specific duplications can occur within the teleost subcluster. The expression data for the labrid transcripts demonstrated that the more ancestral *vtgC *genes that are orthologous to chicken *ggvtgI *are the least expressed and we argue that these minor genes have functionally diverged in the teleost lineage due to loss of the Pv and C-terminal domains. In the closely related family of labrid teleosts, the expression ratios of the major *vtgAb *and neofunctionalized *vtgAa *transcripts reflect the benthic or pelagic character of the spawned egg.

## Methods

### Samples

Mature female cuckoo wrasse (*Labrus mixtus*), rock cook (*Crenilabrus exoletus*) and goldsinny wrasse (*Ctenolabrus rupestris*) were collected using traps and gill nets in the costal waters near Bergen, Norway. Fish were transported live to the laboratory and maintained in fish tanks. Later they where euthanized in accordance with the International Guiding Principles for Biomedical Research Involving Animals as promulgated by the Society for the Study of Reproduction. Subsequent sampling of livers and ovaries was performed in a cold room (4°C). Pre-hydrated oocytes (PH ooc) and ovulated eggs (OV egg) were dissected from the ovaries and processed as described previously [19].

### cDNA cloning

Total RNA was isolated from vitellogenic livers of three rock cook females using RNAeasy kit (Qiagen). Extracts were subsequently mixed together for single strand 3' and 5'-cDNA synthesis using Smart Race cDNA Amplification kit (Clonetech, ). The alignment of Finn & Kristoffersen [4] was used to select areas that were specific to each form of *vtg*. Gene specific primers (GSP) (see Additional file 2) subsequently designed from nt sequences of red seabream *vtgAa*, *vtgAb *and *vtgC *(primers P1, P11 and P21) were then used to run 3' and 5'-RACE polymerase chain reactions (PCR) as recommended by the manufacturer.

A PCR product of approximately 4000 bp was amplified using sense primer P1. It was cloned and sequenced as described previously [19]. Three sense primers (P2–P4) designed from a partial rock cook sequence were used in addition to M13 vector primers to obtain the sequence of the cloned product. In order to sequence the remaining N-terminal area of this gene, a new antisense GSP (P5) was constructed from the aforementioned sequence. The RACE PCR product (~1300 bp) was cloned and bi-directionally sequenced.

An antisense GSP for red seabream *vtgAb *(P11) was used in a 5'-RACE PCR together with single stranded rock cook 5'-cDNA giving ~800 bp long PCR product. After cloning and sequencing, two different ESTs were identified to match the N-terminal end of *vtgAb *in other teleost species using BLAST. To verify that the ESTs represent two novel products, the same experiment was conducted with new total RNAs that were extracted from two females and independently used for single strand cDNA synthesis. PCR products from both reactions were gel-purified, cloned and sequenced giving the same two distinct *vtgAb *sequences. Full sequence of *vtgAb1 *was achieved by primer walking with five sense rock cook GSPs (P12–P16). An additional sense GSP (P17) was used to obtain the remaining part of the partial *vtgAb2 *sequence.

Cloning of *vtgC *was accomplished using an antisense GSP made from red seabream nt sequence (P21) and a sense primer (P22) designed from rock cook ESTs. A PCR product of approximately 3500 bp was amplified using the latter primer and was sequenced with M13 vector primers and three additional sense primers (P23–P25).

The same extraction procedures and sequencing strategies were employed to retrieve full-length sequences of three different *vtg *forms in cuckoo wrasse. Cloning of PCR products amplified with the same red seabream GSPs (P1, P11 and P21) as for rock cook, gave partial sequences that were used to construct new *vtgAa *(P6–P10), *vtgAb *(P18–P20) and *vtgC *(P26–P29) primers. Subsequent PCR reactions, cloning and sequencing of new PCR products were done as described above. Despite using GSPs for both *vtgAb *types in rock cook wrasse (P12 and P13), only a single *vtgAb2 *transcript was obtained.

### Northern blots

Total RNA was extracted from each of the labrid teleost livers, as described above, and electrophoretically fractioned in 1% agarose gels containing formaldehyde (6.7%), stained with ethidium bromide solution to visualize rRNAs and blotted onto a Hybond-N nylon membrane (Amersham) by capillary transfer in 10 × SSC and covalently linked to the membrane by exposure to the UV light. Membranes were prehybridized in hybridization buffer (PerfectHyb™ Plus, Sigma) at 68°C for 60 min before being hybridized with denatured and labeled ^32^P-cDNA probes (Strip-EZ DNA, Ambion) overnight as described previously [53].

Four *vtg *gene specific probes for rock cook and three for cuckoo and goldsinny wrasse ranging is size from 602 to 736 nt were constructed from the N-terminal regions. To verify probe specificity, each was sequenced following amplification, gel purification, and excision. Blots were rinsed two times with 2 × SSC/0.1% SDS for 5 min and once with 1 × SSC/0.1% SDS for 15 min at the room temperature. Additional washing was done with 0.1 × SSC/0.1% SDS twice for 10 min at 68°C. In order to detect the signals, membranes were exposed to Kodak's BioMax MS film for 2 hr.

### Phylogenetic analyses

Multiple sequence alignments of the deduced amino acids were used to generate codon alignments of the sequenced transcripts as described previously [4]. In order to determine gene orthologies, *vtg *genes from each of the currently sequenced teleost genomes were accessed from the ensembl servers (zebrafish, medaka, 3-spined stickleback, torafugu and spotted green pufferfish: ensembl release 49, May 2008). For zebrafish, 8 genes were identified using the graphical view and contiguous alignments of transcripts, of which 2 are located in genbank (see Additional file 1 for accession numbers). For medaka, Babin [7] annoted 5 genes on chromosome 4, however, we only found evidence of 4 *vtg *genes, 3 of which are located between bp 9,868,166 – 9,974,743. A novel construct (*olvtgAa2*) was assembled from transcripts: ENSORLESTT00000013001, ENSORLT00000007668, ENSORLESTT00000012994, ENSORLESTT00000012967, ENSORLESTT00000012953 that encoded a protein of 1671 aa. Similarly, a novel construct for 3-spined stickleback (*gavtgAb*) was assembled from transcripts ENSGACT00000012852 and ENSGACT00000012880 located between bp 12,491,853 – 12,515,240 in group VIII. To provide greater statistical support, the novel sequences from the labrid species were aligned with the genomic variants and other vertebrate taxa for which *vtg *sequences are known (see Additional file 1).

Phylogenetic reconstruction was performed using Bayesian (Mr Bayes 3.1.2: 4 × mcmc chains, 1,000,00 generations, sample frequency 100, burnin 3500; [54], maximum parsimony and neighbour joining (PAUP 4.0b10: 1,000 bootstraps; [55]) analyses of the amino acid and codon alignments, and maximum likelihood analyses of the codon alignments as described by Finn & Kristoffersen [4].

## Authors' contributions

RNF conceived the study, contributed to the experimental design, performed the bioinformatics and drafted the manuscript. JK carried out the molecular experiments, contributed to the experimental design and drafted the manuscript. HK participated in the molecular experiments and contributed to the experimental design. FN participated in the experimental design and edited the manuscript. All authors have read and approved the final manuscript.

## Supplementary Material

Additional file 1 **Accession numbers used in the study.** Non-genomic variants are derived from genbank, while genomic variants for chicken, zebrafish, medaka, torafugu and spotted green pufferfish are taken from ensembl release 49. Click here for file

Additional file 2 **Gene specific primers used in the study.** P1, P11 and P21 are designed from red seabream with accession numbers AB181838, AB181839, AB181840, for *vtgAa, vtgAb*, and *vtgC*, respectively. All other primers are from the Labridae in the study. Click here for file
