# Supplementary material for: Evolution and differential expression of a vertebrate vitellogenin gene cluster
Source: BMC Evol Biol. 2009 Jan 5;9:2. doi: 10.1186/1471-2148-9-2 (PMC2632621; doi:10.1186/1471-2148-9-2)
Supplement: Additional file 1 — Accession numbers used in the study. Non-genomic variants are derived from genbank, while genomic variants for chicken, zebrafish, medaka, torafugu and spotted green pufferfish are taken from ensembl release 49. [file 1471-2148-9-2-S1.pdf]

Title: Accession numbers of the sequences used in the analyses.

Description: Non-genomic variants are derived from genbank, while genomic variants for chicken, zebrafish, medaka, torafugu and spotted green pufferfish are taken from ensembl release 49.

	Animal	Species	Vtg	Accession #
1	Atlantic halibut	*Hippoglossus hippoglossus*	*vtgAa*	ABQ58113
2	Atlantic halibut	*Hippoglossus hippoglossus*	*vtgAb*	ABQ58114
3	Atlantic herring	*Clupea harengus*	*vtgAc1*	FJ441000
4	Atlantic herring	*Clupea harengus*	*vtgAc2*	FJ441001
5	Atlantic herring	*Clupea harengus*	*vtgC*	FJ441002
6	Barfin flounder	*Verasper moseri*	*vtgAa*	AB181833
7	Barfin flounder	*Verasper moseri*	*vtgAb*	AB181834
8	Chicken	*Gallus gallus*	*vtgII*	GGVITIIG
9	Chicken	*Gallus gallus*	*vtgIII*	ENSGALP00000037938
10	Chicken	*Gallus gallus*	*vtgI*	D89547
11	Chinese minnow	*Phoxinus oxycephalus*	*vtgAo1*	EF639845
12	Common carp	*Cyprinus carpio*	*vtgAo2*	AB106873
13	Common carp	*Cyprinus carpio*	*vtgAo1*	AF414432
14	Common mummichog	*Fundulus heteroclitus*	*vtgAa*	U07055
15	Common mummichog	*Fundulus heteroclitus*	*vtgAb*	FHU70826
16	Conger eel	*Conger myriaster*	*vtgAe1*	AB185334
17	Cuckoo wrasse	*Labrus mixtus*	*vtgAa*	FJ456934
18	Cuckoo wrasse	*Labrus mixtus*	*vtgAb2*	FJ456935
19	Cuckoo wrasse	*Labrus mixtus*	*vtgC*	FJ456936
20	Fathead minnow	*Pimephales promelas*	*vtgAo1*	AF130354
21	Galaxy coral	*Galaxea fascicularis*	*vtg*	AB179781
22	Goldsinny wrasse	*Ctenolabrus rupestris*	*vtgAa*	EU011580
23	Goldsinny wrasse	*Ctenolabrus rupestris*	*vtgAb1*	EU011581
24	Goldsinny wrasse	*Ctenolabrus rupestris*	*vtgAb2*	FJ456929
25	Goldsinny wrasse	*Ctenolabrus rupestris*	*vtgC*	EU011582
26	Gray Mullet	*Mugil cephalus*	*vtgAa*	AB288932
27	Gray Mullet	*Mugil cephalus*	*vtgAb*	AB288933
28	Gray Mullet	*Mugil cephalus*	*vtgC*	AB288934
29	Green spotted puffer	*Tetraodon nigriviridis*	*vtgAa*	GIDT00021869001
30	Green spotted puffer	*Tetraodon nigriviridis*	*vtgAb*	GIDT00021869001
31	Green spotted puffer	*Tetraodon nigriviridis*	*vtgC*	GSCT00019545001
32	Günther´s walking catfish	*Clarias macrocephalus*	*vtgAo1*	EU138884
33	Haddock	*Melanogrammus aeglefinus*	*vtgAa*	AF284035
34	Haddock	*Melanogrammus aeglefinus*	*vtgAb*	AF284034
35	Japanese eel	*Anguilla japonica*	*vtgAe1*	AY775788
36	Japanese eel	*Anguilla japonica*	*vtgAe2*	AY423445
37	Japanese eel	*Anguilla japonica*	*vtgAe3*	AY423444
38	Medaka	*Oryzias latipes*	*vtgAa1*	AB064320
39	Medaka	*Oryzias latipes*	*vtgAa2*	ENSORLESTP00000013001-7667-12994-12967-12953
40	Medaka	*Oryzias latipes*	*vtgAb*	AB074891
41	Medaka	*Oryzias latipes*	*vtgC*	ENSORLP00000008173
42	Mosquitofish	*Gambusia affinis*	*vtgAa*	AB181835
43	Mosquitofish	*Gambusia affinis*	*vtgAb*	AB181836
44	Mosquitofish	*Gambusia affinis*	*vtgC*	AB181837
45	Rainbow trout	*Oncorhynchus mykiss*	*vtgAsa*	X92804
46	Red seabream	*Pagrus major*	*vtgAa*	AB181838
47	Red seabream	*Pagrus major*	*vtgAb*	AB181839
48	Red seabream	*Pagrus major*	*vtgC*	AB181840
49	Rock cook	*Centrolabrus exoletus*	*vtgAa*	FJ456930
50	Rock cook	*Centrolabrus exoletus*	*vtgAb1*	FJ456931
51	Rock cook	*Centrolabrus exoletus*	*vtgAb2*	FJ456932
52	Rock cook	*Centrolabrus exoletus*	*vtgC*	FJ456933
53	Silver lamprey	*Ichthyomyzon unicuspus*	*vtg*	M88749
54	Three-spined stickleback	*Gasterosteus aculeatus*	*vtgAa*	ENSGACP00000012923
55	Three-spined stickleback	*Gasterosteus aculeatus*	*vtgAb*	ENSGACP00000012828-12856
56	Three-spined stickleback	*Gasterosteus aculeatus*	*vtgC*	ENSGACP00000012536
57	Torafugu	*Takifugu rubripes*	*vtgAa*	ENSTRUG00000017475
58	Torafugu	*Takifugu rubripes*	*vtgAb*	ENSTRUG00000017475
59	Torafugu	*Takifugu rubripes*	*vtgC*	ENSTRUP00000009572
60	White cloud mountain minno	*Tanichthys albonubes*	*vtgAo1*	EF370398
61	White perch	*Morone americana*	*vtgAa*	AAZ17415
62	White perch	*Morone americana*	*vtgAb*	AAZ17416
63	White perch	*Morone americana*	*vtgC*	AAZ17417
64	White sturgeon	*Acipenser transmontanus*	*vtg*	U00455
65	Whitefish	*Coregonus lavaretus*	*vtgAsb*	AF454745
66	Whitefish	*Coregonus lavaretus*	*vtgAsb*	AF454746
67	Zebrafish	*Danio rerio*	*vtg1 (Ao1)*	AF406784
68	Zebrafish	*Danio rerio*	*vtg4 (Ao1)*	ENSDARP00000072674
69	Zebrafish	*Danio rerio*	*vtg5 (Ao1)*	ENSDARP00000061222
70	Zebrafish	*Danio rerio*	*vtg6 (Ao1)*	ENSDARP00000095999
71	Zebrafish	*Danio rerio*	*vtg7 (Ao1)*	ENSDARP00000072678
72	Zebrafish	*Danio rerio*	*vtg8 (Ao2)*	ENSDARP00000072780
73	Zebrafish	*Danio rerio*	*vtg2 (Ao2)*	ENSDARP00000061164
74	Zebrafish	*Danio rerio*	*vtg3 (C)*	AF254638
