# Supplementary material for: Evolution and differential expression of a vertebrate vitellogenin gene cluster
Source: BMC Evol Biol. 2009 Jan 5;9:2. doi: 10.1186/1471-2148-9-2 (PMC2632621; doi:10.1186/1471-2148-9-2)
Supplement: Additional file 2 — Gene specific primers used in the study. P1, P11 and P21 are designed from red seabream with accession numbers AB181838, AB181839, AB181840, for vtgAa, vtgAb, and vtgC, respectively. All other primers are from the Labridae in the study. [file 1471-2148-9-2-S2.pdf]

Title: Gene specific primers used in the study.

Description: P1, P11 and P21 are designed from red seabream with accession numbers AB181838, AB181839, AB181840, for *vtgAa, vtgAb,* and *vtgC,* respectively. All other primers are from the Labridae in the study.

	primer	Sequence
vtgAa	P1	5’-GCAGACCAAGCAATCCTTGGTGTTCCTTGA-3’
	P2	5’-TGCCCTGTCGAGCTTATAAAGC-3’
	P3	5’-GTTGCAAGACTTGACATCAAGGAG-3’
	P4	5’-CTTGGCTTGGAGCATACACAGA-3’
	P5	5’-TCAGAGCAGCAGAAGTCCCAGTGGCAGGAA-3’
	P6	5’-CGACAGGGCAGACAGGCAAATCGGTGCAAT-3’
	P7	5’-TGCACCGATTTGCCTGTCTGCCCTGTCGA-3’
	P8	5’- ATCCTGCCCATCCATGGCACAGCAGCTACA-3’
	P9	5’- CAACGAGGGCAACTTTAAGGTC -3’
	P10	5’- CAGGTCGATATCAATGTCAGCAA -3’
vtgAb	P11	5’-CATCTGGGCAGCGCCATTCAAGATGTTGA-3’
	P12	5’-TCACCTCTGCCCTGGCTGCTCAGCTCTTGA-3’
	P13	5’-TTCTTCGGACAGGAAATTGCATTTGCCAACA-3’
	P14	5’-AGACCTTGCAGCAGCCAAATTCACACCGAT -3’
	P15	5’- GCATCAGCCAGAGTAGTCACCA-3’
	P16	5’- GCGGAGAACGACCACTATAGGA -3’
	P17	5’-TGCGTGGAGTGCGAGGCTAGAGGAAAGTCT-3’
	P18	5’- TTGAGACCAAGCTGCCAATGGGTTTGGTGA -3’
	P19	5’- GGGCCAGTCATTGAGAAGATTG -3’
	P20	5’- AACTGAGTGTTGCTGTTGCTTCTG -3’
vtgC	P21	5’-TGGTACTGGGTCATCCAGGTTCTGCATCAT-3’
	P22	5’-CAAACCAACGGCAGAGGGAGGTGTCATTACA-3’
	P23	5’- TTCTCCCTGGAGTCTCTGCCAC -3’
	P24	5’- AGCCTCCTCAAAGTCTCAAGCAG -3’
	P25	5’- ACATCCAGATAGTGGTCGCCAA -3’
	P26	5’- ATGGTGCTGCTTGGGGTGACTGACACAGCT -3’
	P27	5’- TTTCCCTGGTGAAGGACTGACAG -3’
	P28	5’- GTTCATAAAGGACCTAATTGCGG -3’
	P29	5’- GTGAAGGGCGGTAATTTCAAGATGCAAGCAA -3’
